# *In Situ* Engineering of Two-Dimensional Heterostructures for Enhanced Photocatalytic Decontamination of Methyl Orange

**DOI:** 10.1021/acsaenm.5c00134

**Published:** 2025-05-14

**Authors:** Junli Chen, Xinyi Jin, Pengcheng Zhang, Nan Song, Pan Gao, Roland A Fischer, Soumya Mukherjee

**Affiliations:** † College of Materials and Chemical Engineering, Collaborative Innovation Center of Environmental Pollution Control and Ecological Restoration, 117776 Zhengzhou University of Light Industry, Zhengzhou 450002, P. R. China; ‡ Chair of Inorganic and Metal−Organic Chemistry, Department of Chemistry, School of Natural Sciences and Catalysis Research Center, 686517 Technische Universität München, Lichtenbergstraße 4, Garching b. München 85748, Germany; § Department of Chemical Sciences, Bernal Institute and Research Ireland Centre for Pharmaceuticals (SSPC), 8808 University of Limerick, Limerick V94 T9PX, Ireland

**Keywords:** 2D materials, p−n heterojunction, in situ synthesis, environmental remediation, photocatalysis

## Abstract

Two-dimensional (2D)/2D heterostructured catalysts have garnered significant attention in photocatalytic environmental remediation due to their relevance in optoelectronic, as well as solar energy conversion systems. However, fast photocarrier separation of 2D/2D heterojunctions, made from the stacking of different layered materials through strong chemical bonds rather than weak van der Waals interactions, remains an unmet challenge. To address this, herein, a generation of 2D/2D p–n heterojunction photocatalysts, composed of BiOCl and HMo_*x*_Nb_3__–*x*_O_8_ nanosheets, was fabricated via *in situ* chemical absorption and hydrolysis strategies. This close heterojunction interface enhanced the separation and migration of photoinduced electron–hole (e^–^–h^+^) pairs. As a result, the prepared ultrathin BiOCl/HMo_*x*_Nb_3–*x*_O_8_ heterostructure catalysts demonstrated superior photocatalytic degradation of methyl orange (MO) under UV–visible light, with the optimized photocatalysts (BiOCl/NbMo_–10_) achieving MO removal efficiencies 2.94 and 2.22 times greater than those of pristine BiOCl and NbMo_–10_ materials, respectively. Further, hydroxyl radicals (·OH), positive holes (h^+^), and superoxide anions (·O_2_^–^) were also confirmed to play key roles in MO removal within the photocatalytic system. This work offers insights into the rational design and *in situ* construction of high-performance 2D/2D heterojunction photocatalysts for environmental remediation.

## Graphical abstract

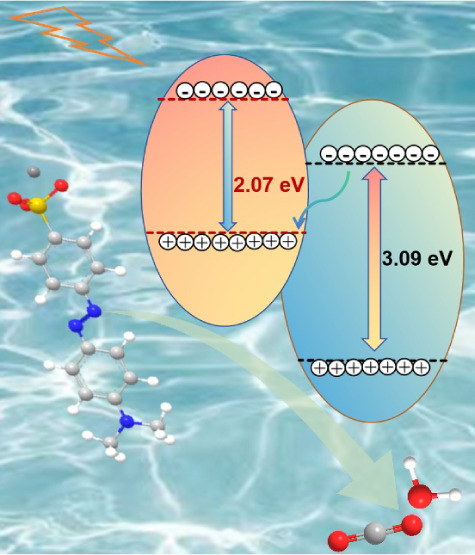

## 1 Introduction

Environmental pollution is a pressing global challenge, severely disrupting ecosystems and endangering human health as a direct consequence of accelerated urbanisation and industrial growth.^1,2^ Developing efficient, environmentally friendly remediation methods is therefore critical. Photocatalytic degradation, a clean and resource-efficient technique, has gained attention for converting pollutants into harmless species under light irradiation.^3–6^ Advances in materials engineering have positioned two-dimensional (2D) semiconductor photocatalysts as promising candidates for environmental purification, thanks to their enhanced photoelectrochemical properties and the integration of semiconducting behaviors with photochemical catalysis.^7–9^ Among these, n-type niobium oxide composite nanosheets stand out due to their unique photoelectronic properties, excellent photocatalytic activity, and layered structures, which expose active sites and improve mass and electron transfer. For instance, Zhang et al. reported that 2D HNb_3_O_8_ nanosheets featured broad, smooth surfaces that facilitated the exposure of active sites, thereby promoting efficient migration of photogenerated charge carriers under UV excitation and generating highly reactive free radicals (e.g., −OH and −O_2_^–^) to enhance photocatalytic performance.^10^ These materials have shown promise in photocatalytic oxidation, water splitting, molecular separation, and CO_2_ reduction.^11,12^ However, their photocatalytic efficiency is often handicapped by inadequate separation and rapid recombination of photogenerated electron–hole (e^–^–h^+^) pairs during photoreactions. Although nonprecious metal doping has been extensively explored to enhance charge separation by modulating the electronic structures of photocatalysts,^13,14^ the intrinsic semiconductive properties of metal-doped niobium materials still result in inefficient light harvesting and charge separation, limiting their applications. Heterojunction engineering through morphological control and composite coupling in the design of photocatalysts has drawn increasing attention for enhancing charge separation and photocatalytic performance.^15,16^ The formation of p–n heterojunctions with an effective built-in electric field has emerged as a promising approach to improve light absorption and the separation of photogenerated electron–hole (e^–^–h^+^) pairs in photocatalysts.^17–20^ Consequently, introducing a suitable p-type semiconductor to n-type niobium materials to construct p–n heterojunctions is essential for enhancing photocatalytic performance.

Bismuth oxychloride (BiOCl), a layered p-type semiconductor that offers an ca. 3.4 eV band gap, not only exhibits excellent solar light absorption and utilization, but can also match well with semiconductors for enhancing the charge separation ability.^21,22^ For example, Yuan et al. constructed Cu_2_O/BiOCl heterojunction with high photocatalytic performance for tetracycline degradation.^23^ Gupta and Kansal designed Bi_3_O_4_Cl/BiOCl composites to realize levofloxacin removal efficiently.^24^ However, those conventional stacks of different semiconductors together via van der Waals forces are still limited by weak interactions at the heterojunction interface and reduced electron transfer efficiency.^25,26^ Chemical bonding between different material layers can not only enhance photoexcited carrier separation but also even induce new energy band structure, which could greatly improve light harvest and charge separation. It is essential, therefore, to design and construct p–n heterojunctions via chemical bonds with strong interfacial interactions and short charge migration pathways. Notably, BiOCl exhibits a highly anisotropic sandwich structure, with [Bi_2_O_2_]^2+^ layers intercalated between two layers of Cl^–^, affording a large contact interface that facilitates the chemical adsorption of Bi and Cl species. This structure can promote the formation of high-contact heterostructures on the surfaces of other materials.^27^ Previous studies have shown that Bi^3+^ ions can first adsorb onto substrates and subsequently act as bonding sites for Cl^–^ ions, leading to the formation of BiOCl sheets.^28,29^ This insight inspired the construction of p–n heterostructured photocatalysts by growing BiOCl *in situ* on n-type niobium materials. Leveraging the close bandgap alignment between BiOCl and HNb_3_O_8_, BiOCl grown chemically *in situ* on the surface of molybdenum-doped niobium oxide nanosheets (BiOCl/HMo_*x*_Nb_3–*x*_O_8_) is expected to enhance electron–hole (e^–^–h^+^) separation, thereby profoundly impacting photocatalytic performance.

Herein, we report on a new platform of 2D/2D p–n heterojunction photocatalysts comprising BiOCl and HMo_*x*_Nb_3–*x*_O_8_ nanosheets using *in situ* chemical absorption and hydrolysis strategies (Scheme 1). The resulting BiOCl/HMo_*x*_Nb_3–*x*_O_8_ heterostructure catalysts demonstrated enhanced performance for the photodegradation of methyl orange (MO) under UV–visible (UV–vis) light. The optimized photocatalyst, BiOCl/NbMo_–10_ revealed superior MO degradation efficiencies of 2.94 and 2.22 times compared to pristine BiOCl and NbMo_–10_. This improvement is attributed to the synergistic effects of tightly integrated p–n heterojunctions formed via the *in situ* synthesis method and the improved photoinduced e^–^–h^+^ separating and transferring efficiency. Further, potential photocatalytic mechanisms for MO degradation and the roles of the primary active species (·OH, h^+^, and ·O_2_^–^) were proposed. This work provides valuable insights into the design and construction of high-performance 2D/2D heterojunction photocatalysts for environmental remediation applications.

**Scheme 1:**
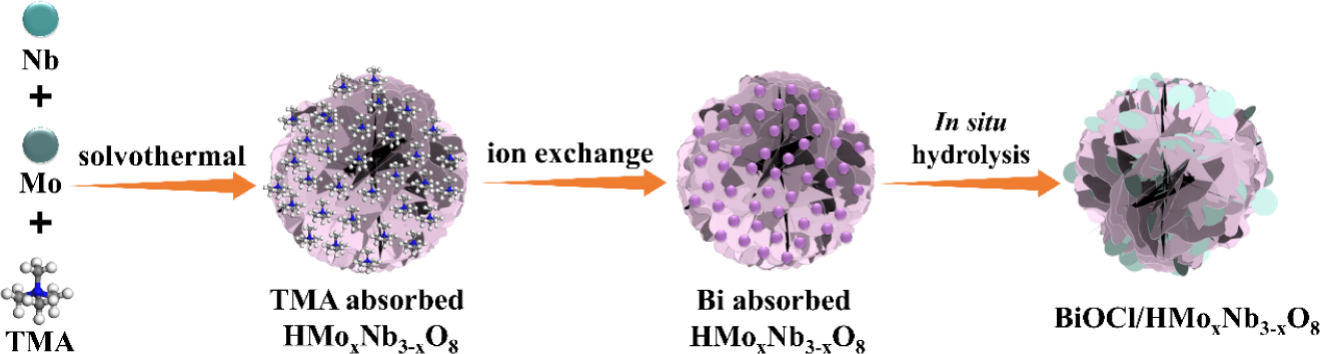
Schematic Presenting the *In Situ* Synthesis of BiOCl/HMo_*x*_Nb_3–*x*_O_8_ Photocatalysts

## 2 Experimental Section

### 2.1 Materials

Niobium chloride (NbCl_5_, 99.9%), molybdenum pentachloride (MoCl_5_, 99.6%), and tetramethylammonium hydroxide solution (TMAOH, weight (wt.) 25%) were obtained from Aladdin. Methyl orange was bought from Merck. Ethanol (EtOH, 99.7%, AR) was provided by Tianjin Kermel Chemical Reagent Co., Ltd., and deionized water (H_2_O, 18.25 MΩ) was utilized for all experiments.

### 2.2 Preparation of BiOCl/HMo_*x*_Nb_3–*x*_O_8_ Materials

#### 2.2.1 Synthesis of HMo_*x*_Nb_3–*x*_O_8_

HMo_*x*_Nb_3–*x*_O_8_ nanosheets were synthesized solvothermally. A total of 0.81 g of niobium pentachloride (NbCl_5_, 3 mmol) and 0.082 g of molybdenum pentachloride (MoCl_5_, 0.3 mmol) were dissolved in 18 mL of anhydrous ethanol. Subsequently, 10 mL of TMAOH and 16 mL of H_2_O were incrementally added to the above solution under continuous stirring at a flow rate of 0.6 mL·min^–1^. Once the addition of TMAOH was complete, the resulting clear solution was left at room temperature for 2 h. The mixture was then transferred into a 100 mL stainless steel autoclave with a Teflon liner and treated at 240 °C for 12 h. Finally, the HMo_*x*_Nb_3–*x*_O_8_ nanosheets were obtained by filtering and rinsing the resulting products with ethanol and water, followed by vacuum drying at 60 °C for 8 h. These nanosheets were designated as NbMo_–10_. Different HMo_*x*_Nb_3–*x*_O_8_ materials, designated as NbMo_–5_ and NbMo_–12_, were synthesized by adjusting the molar percentage of MoCl_5_ to NbCl_5_ to 5% and 12%, respectively.

#### 2.2.2 *In Situ* Synthesis of BiOCl/HMo_*x*_Nb_3–*x*_O_8_

First, 1 g of the prepared NbMo_–10_ materials was introduced into 24 mL of 0.5 M HCl solution containing 0.946 g of BiCl_3_. Following 6 h of continuous stirring and an additional 8 h undisturbed at room temperature, after thorough rinsing with H_2_O and drying at 60 °C for 8 h, BiOCl/NbMo_–10_ materials were synthesized *in situ*. BiOCl/NbMo_–5_ and BiOCl/NbMo_–12_ were synthesized by changing NbMo_–10_ to NbMo_–5_ and BiOCl/NbMo_–12_, respectively.

#### 2.2.3 Physical Mixing-Based Synthesis of BiOCl/HMo_*x*_Nb_3–*x*_O_8_

A total of 5 mg of NbMo_–10_ and 15 mg of commercial BiOCl were added to 20 mL of H_2_O and stirred at room temperature for 6 h. The resulting products were collected via centrifugation and dried at 60 °C for 8 h. This material was designated as BiOCl/NbMo_–10‑s_.

### 2.3 Material Characterization

Powder X-ray diffraction (PXRD) patterns were recorded using a Bruker D8 ADVANCE X-ray diffractometer over a 2θ range of 5° to 80°, with monochromatized Cu Kα radiation. The size and morphology of the materials were observed via field emission scanning electron microscopy (FESEM, JSM-7001F, Japan) at an operating voltage of 5–30 kV. High-resolution transmission electron microscopy (HRTEM, JEM-2100, Japan), operating at 200 kV and providing magnifications of 6,000× to 600,000×, was employed to analyze the microstructural and crystallographic features of the specimens. The light absorption capabilities and electronic band structure were investigated using UV–vis diffuse reflectance spectroscopy with a Hitachi U-3900 spectrophotometer (Japan) over the wavelength range of 250–800 nm, with a slit width of approximately 0.5 nm and a scanning rate of about 120 nm min^–1^. The optical absorption properties of the samples were evaluated using the same UV–vis diffuse reflectance spectroscopy setup. Electron paramagnetic resonance (EPR) spectra were recorded using a Bruker A300 spectrometer (Germany) to assess the generation of radicals, with 5,5-dimethyl-1-pyrroline-*N*-oxide (DMPO) and 2,2,6,6-tetramethyl-1-piperidinyloxy (TEMPO) serving as spin-trap agents.

### 2.4 Photocatalytic Degradation of Environmental Contaminants

The photocatalytic performance of BiOCl/HMo_*x*_Nb_3–*x*_O_8_ was evaluated by monitoring the decolorization efficiency of MO in an aqueous medium. For the experimental setup, a 300 W xenon (Xe) lamp was used as the simulated solar light source. Typically, 20 mg of photocatalyst was added to a 100 mL beaker containing 50 mL of a 10 mg/L MO solution. Initially, the mixture was stirred continuously in the dark for 30 min to achieve adsorption–desorption equilibrium. During the photodegradation process, the MO solution containing the photocatalyst was continuously stirred with a Teflon-coated magnetic bar while being exposed to simulated solar irradiation. At specific intervals, 3 mL of above solution was withdrawn, and the MO concentration was measured using a UV–vis spectrometer (Hitachi, U-3900H).

### 2.5 Photoelectrochemical Measurements

Transient photocurrent responses and electrochemical impedance spectroscopy (EIS) were recorded on a CHI660e electrochemical analyzer (Shanghai Chen Hua Instruments) in a standard three-electrode configuration, with a 0.1 mol L^–1^ Na_2_SO_4_ aqueous solution serving as the electrolyte. A film of BiOCl/HMo_*x*_Nb_3–*x*_O_8_ was prepared by depositing 200 μL of BiOCl/HMo_*x*_Nb_3–*x*_O_8_ ink (1 mg·mL^–1^ in H_2_O) onto fluorine-doped tin oxide (FTO) glass (fixed area: 1 cm^2^), which was used as the working electrode after dried at 60 °C for 4 h. A platinum plate and an Ag/AgCl electrode were used as the counter electrode and reference electrode, respectively. A 300 W Xe lamp was employed as the light source. EIS measurements were conducted using an alternating current amplitude of 10 mV across a frequency range of 0.1 Hz to 100 kHz. Mott–Schottky analyses were carried out under a 10 mV amplitude at a frequency of 1 kHz.

## 3 Results and Discussion

### 3.1 Characterization of BiOCl/HMo_*x*_Nb_3–*x*_O_8_ Materials

PXRD patterns were recorded to investigate the compositions of the prepared polycrystalline materials. As shown in Figure 1A, the characteristic diffraction peaks of the prepared NbMo_–10_ materials were found to be consistent with those in H_3_ONb_3_O_8_ (PDF #44-0672), indicating no structural changes to the niobium oxides upon Mo-doping.^30,31^ The diffraction peaks located at 12.00°, 24.13°, 25.88°, 32.5°, 33.47°, 36.56°, and 40.91° correspond to (001), (002), (101), (102), (003), and (112) planes of BiOCl, respectively.^32^ The higher peak intensities for NbMo_–10_ in the BiOCl/NbMo_–10_ materials indicated higher crystallinity and larger particle size compared to pristine NbMo_–10_. Absence of any other diffraction peaks across all samples reinforced high phase purity for the synthesized materials studied herein.

**Figure 1:**
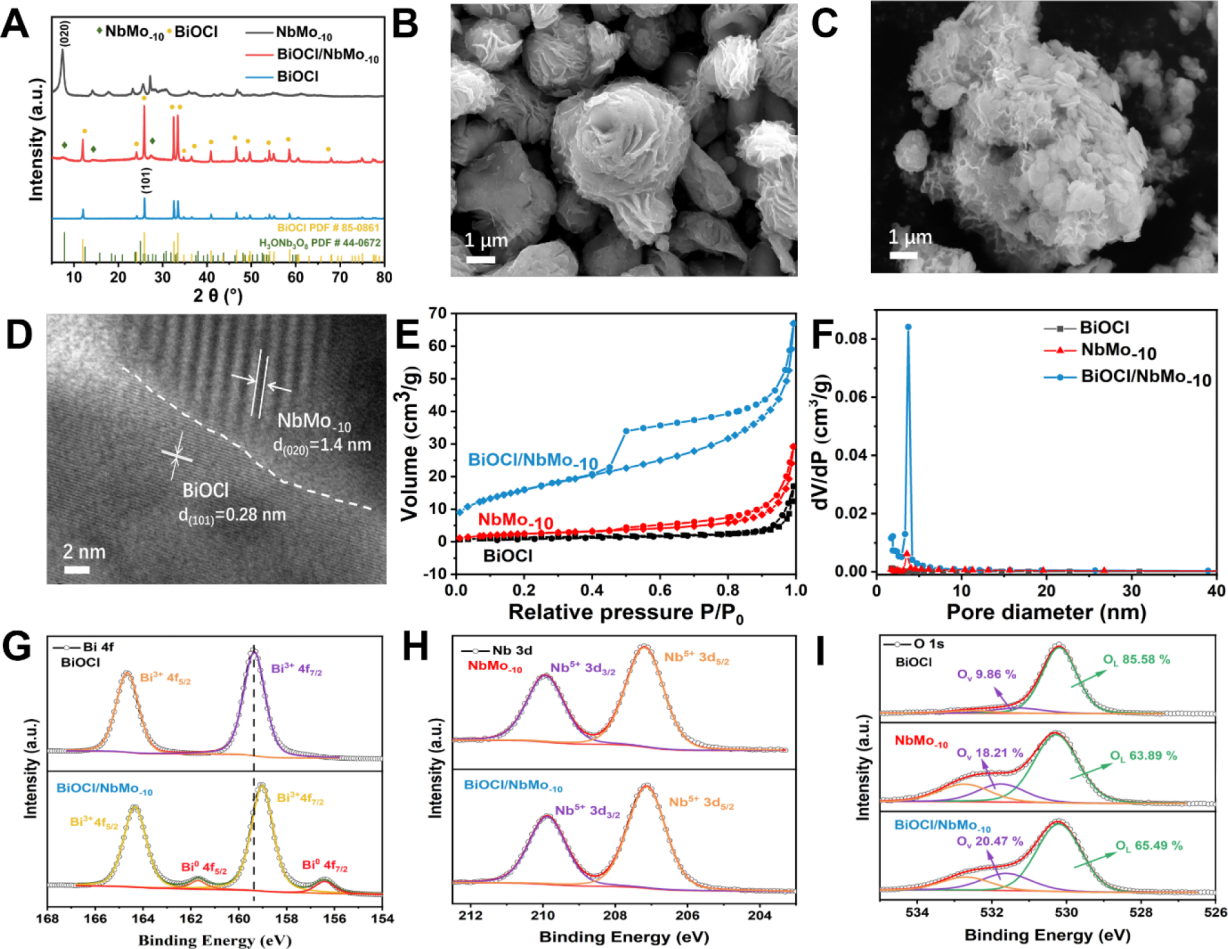
(A) PXRD patterns of NbMo_–10_, BiOCl, and BiOCl/NbMo_–10_. SEM micrographs of (B) NbMo_–10_, (C) BiOCl/NbMo_–10_. (D) HRTEM image of BiOCl/NbMo_–10_. (E) N_2_ adsorption–desorption isotherms of BiOCl, NbMo_–10_, and BiOCl/NbMo_–10_ materials. (F) Pore size distribution profiles of BiOCl, NbMo_–10_, and BiOCl/NbMo_–10_, high-resolution X-ray photoelectron spectra of Bi 4f (G), Nb 3d (H) and O 1s (I).

Scanning electron microscopy (SEM) micrographs were employed to examine the morphologies of the prepared NbMo_–10_ and BiOCl/NbMo_–10_ materials. As shown in Figure 1B, NbMo_–10_ revealed three-dimensional (3D) spherical flower-like structures assembled from nanosheets with an edge thickness of ca. 51.2 nm. These self-assembled nanosheets exhibited a propensity to form organized flower-like superstructures rather than random aggregates, likely driven by van der Waals forces. These superstructures, rather than random aggregates, provided larger surface areas and exposed more active sites, facilitating oxidation reactions and improving charge transport efficiency across the surface. Besides, as shown in Figure S1A, BiOCl/NbMo_–10_ retained the same flower-like structure as NbMo_–10_ prior to the hydrolysis treatment, suggesting that [Bi_2_O_2_]^2+^ species were chemically absorbed without forming BiOCl on the NbMo_–10_ surface. After hydrolysis, Figures 1C and S1B reveal that 2D BiOCl sheets, with an average thickness of 38.1 nm, were integrated onto the NbMo_–10_ surface, resulting in a confined 2D/2D heterostructure. The close interaction between the NbMo_–10_ petals and BiOCl nanosheets reduced the travel distance of photoinduced electron–hole (e^–^–h^+^) pairs, thereby enhancing the photocatalytic performance of the BiOCl/NbMo_–10_ materials. Transmission electron microscopy (TEM) and high-resolution transmission electron microscopy (HRTEM) were employed to further examine the morphology and microstructure of the BiOCl/NbMo_–10_ material. As shown in Figure S2, BiOCl nanosheets were clearly dispersed on the surface of the 2D NbMo_–10_ thin nanosheets, consistent with the SEM results. Additionally, lattice fringes corresponding to NbMo_–10_ and BiOCl were readily observed in Figure 1D. The lattice spacings of 1.4 and 0.28 nm were attributed to the (020) plane of NbMo_–10_ and the (101) plane of BiOCl, respectively. A distinct boundary between the two phases, NbMo_–10_ and BiOCl, further confirmed the formation of the BiOCl/NbMo_–10_ heterojunction. This intimate contact indicates efficient charge migration between the two phases, enhancing their functionality. The surface area and pore properties of nanomaterials play a key role in molecular absorption and mass diffusion during the MO removal process.

N_2_ adsorption–desorption isotherms were recorded to investigate the surface areas and pore size characteristics of prepared various photocatalysts. Figure 1E,F exhibited the type IV isotherms and the pore size distributions of all three materials (BiOCl, NbMo_–10_, and BiOCl/NbMo_–10_), respectively. The Brunauer–Emmett–Teller (BET) specific surface areas, pore volumes, and average pore sizes were determined using the Barrett–Joyner–Halenda (BJH) method and are summarized in Table S1. Compared to the surface areas of NbMo_–10_ (9.35 m^2^·g^–1^) and BiOCl (4.97 m^2^·g^–1^), the surface area of the *in situ* synthesized BiOCl/NbMo_–10_ was significantly increased to 58.07 m^2^·g^–1^, this enhancement promotes mass diffusion and provides more active sites during photocatalysis. The presence of mesopores was further beneficial to the mass transfer and diffusion. Notably, the reduced pore size of BiOCl/NbMo_–10_ also confirms the formation of heterostructures, aligning well with the SEM and TEM results.

X-ray photoelectron spectroscopy (XPS) was employed to investigate changes in the surface composition and chemical states of the synthesized materials. As shown in Figure S3A, the presence of Bi, O, Cl, Nb, and Mo elements in BiOCl/NbMo-10 confirms the successful formation of the composite. High-resolution XPS spectra of Bi 4f for both BiOCl and BiOCl/NbMo-10 are presented in Figure 1G. The characteristic peaks at 164.65 and 159.35 eV in pristine BiOCl correspond to Bi 4f_5/2_ and 4f_7/2_, respectively. The spin–orbit splitting energy of 5.3 eV is consistent with the trivalent oxidation state of Bi^3+^ in the crystal structure.^33^ In the case of BiOCl/NbMo_–10_, the Bi 4f peaks exhibit a clear shift toward lower binding energies, likely due to the presence of oxygen vacancies (OVs) leading to undercoordinated Bi^3+^ sites near defect regions. These vacancies may facilitate electron transfer to Bi centers, thereby reducing their local coordination and lowering the observed binding energies. The emergence of Bi^0^ species is possibly attributable to the accumulation of photogenerated electrons through localization and the inevitable self-reduction process during synthesis.^34^ Furthermore, a slight positive shift in Nb 3d_5/2_ binding energy is observed, from 209.36 eV in NbMo_–10_ to 209.88 eV in BiOCl/NbMo_–10_ (Figure 1H), suggesting enhanced covalency in the Nb–O bond. This could be associated with the formation of Nb–O–Bi bonds at the heterojunction interface, facilitating electron transfer from NbMo_–10_ to BiOCl. Meanwhile, the binding energy of Mo 3d (Figure S3C) shows a negative shift of 0.4–0.6 eV, indicative of a partial reduction of Mo species (Mo^6+^ → Mo^5+^). This suggests a potential role for Mo as an electron transport medium, aiding the separation and migration of photogenerated carriers.^35^ As depicted in Figure 1I, the O 1s spectra show a notable increase in oxygen vacancy content in BiOCl/NbMo_–10_ (531.62 eV, 20.47%) compared to NbMo_–10_ (18.21%), possibly due to oxygen atom desorption caused by lattice mismatch at the interface.^36^ Additionally, the reduction of Mo^6+^ to Mo^5+^ may further contribute to oxygen vacancy formation through lattice oxygen loss, supporting the discussion above. All things considered, the shifts in binding energy clearly confirm the strong electronic coupling at the BiOCl/NbMo_–10_ heterojunction interface. The Mo species functioned as electron transfer channels, facilitating the injection of electrons from NbMo_–10_ into BiOCl. This, in turn, led to the partial reduction of Bi^3+^ and the synergistic formation of oxygen vacancies (VOs). Such multiscale interactions effectively modulated the energy bands of the heterostructure. Moreover, the VOs served as electron trapping centers, helping to prolong carrier lifetimes, while the dual presence of Bi^0^ species and Mo^5+^ contributed to the formation of bimetallic active sites. This interplay between the tuned electronic properties and the built-in electric field at the heterojunction underpins the improved photocatalytic reduction performance.

The optical absorption properties of BiOCl, NbMo_–10_, and BiOCl/NbMo_–10_ were examined using UV–vis spectroscopy. As shown in Figure S5, a notable red shift and visible absorption were observed in the BiOCl/NbMo_–10_ composites compared to pure BiOCl, which was primarily attributed to the excellent optical absorption of NbMo_–10_ and the synergistic interaction between BiOCl and NbMo_–10_. Tauc plots predicated upon the formula of *αhv* = *A*(*hv* – *E*_g_)^*n*/2^ (*α h, ν, E*_g_, and *A* mean absorption coefficient, Planck constant, light frequency, band gap energy, and a constant, *n* is set to 1/2 for indirect band gap materials) were used to calculate the band gap energy (*E*_g_) of synthesized indirect semiconductors.^37^ From the plot of (*αhv*)^2^ versus (*hv*) presented in Figure 2A, *E*_g_ values for BiOCl, NbMo_–10_ and BiOCl/NbMo_–10_ were identified as 3.09, 2.07, and 2.81 eV, respectively. The lowest *E*_g_ value for BiOCl/NbMo_–10_ corresponded to its highest optical absorption, as shown in Figure S5.

**Figure 2:**
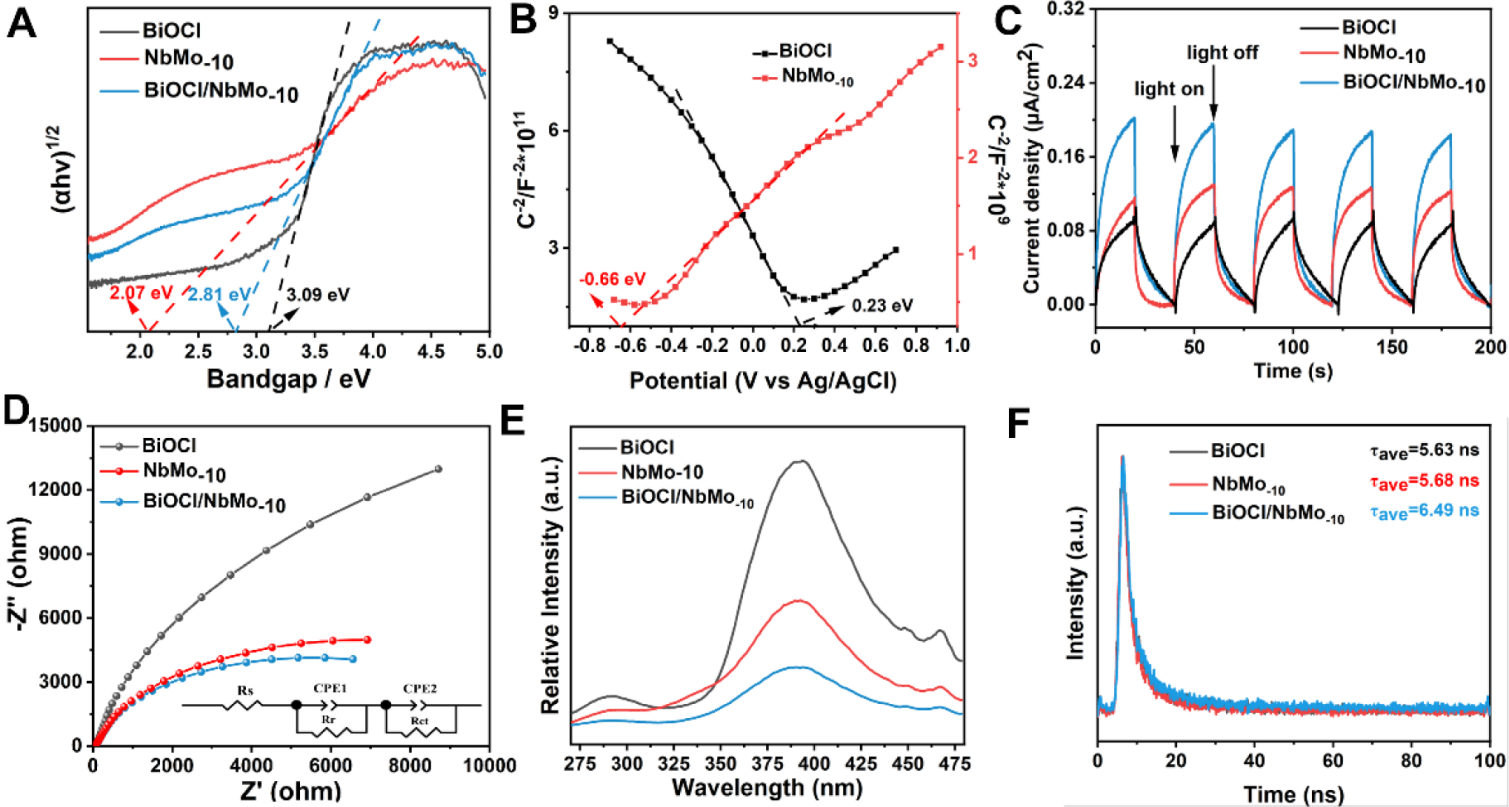
(A) Tauc curves of BiOCl, NbMo_–10_ and BiOCl/NbMo_–10_. (B) Mott–Schottky plots of BiOCl and NbMo_–10_. (C) Photocurrent responses of BiOCl, NbMo_–10_ and BiOCl/NbMo_–10_. (D) EIS Nyquist plots of BiOCl, NbMo_–10_ and BiOCl/NbMo_–10_ at open circuit voltage. (E) Photoluminescence spectra of BiOCl, NbMo_–10_ and BiOCl/NbMo_–10_, (F) time-resolved photoluminescence spectra of prepared photocatalysts.

Mott–Schottky plots were further recorded to determine the semiconductor type and energy band configuration of BiOCl and NbMo_–10_. The negative slope and the positive slope in Figure 2B indicated that BiOCl and NbMo_–10_ were p-type and n-type semiconductors, respectively, consistent with previous reports.^12,38,39^ Thanks to these results, it can be inferred that the effective utilization of incident light, combined with the synergistic effect of the 2D/2D p–n heterojunction, enhances the photocatalytic activity of BiOCl/NbMo_–10_. Additional UV–vis absorption spectra of BiOCl/HMo_*x*_Nb_3–*x*_O_8_ photocatalysts (BiOCl/NbMo_–5_, BiOCl/NbMo_–10_, and BiOCl/NbMo_–12_) are shown in Figure S6, revealing an obvious red shift in the absorption spectra with increasing Mo doping from NbMo_–5_ to NbMo_–10_. However, a slight blue shift with further Mo doping was observed, likely due to the formation of impurities, such as MoO_3_, from excess Mo.^40^ Therefore, optimized Mo doping was found to be beneficial for enhancing light adsorption and improving the photocatalytic performance of these optical materials. Photocurrent responses and EIS were conducted to assess the separation efficiency of photoinduced electron–hole pairs (e^–^–h^+^) and the electron transfer dynamics.^41–43^ As shown in Figure 2C, BiOCl/NbMo_–10_ exhibited the highest photocurrent responses compared to pristine BiOCl and NbMo_–10_, indicating enhanced electron transfer capabilities in the p–n heterostructured BiOCl/NbMo_–10_ materials under irradiation.^44^

The EIS profile presented in Figure 2D provided insights into the charge transfer dynamics at the electrode–electrolyte interface. The semicircular arc observed corresponded to the charge transfer resistance (*R*_ct_), where a larger diameter indicated slower charge transfer kinetics at the interface. From Figure 2E, BiOCl/NbMo_–10_ demonstrated the smallest *R*_ct_ compared to NbMo_–10_ and BiOCl (Table S2), signifying the lowest charge transfer resistance and the highest conductivity. Photocurrent responses and EIS spectra of other BiOCl/HMo_*x*_Nb_3–*x*_O_8_ materials, not discussed here, are shown in Figure S8. The markedly enhanced photocurrent response and reduced charge transfer resistance observed in BiOCl/NbMo_–10_ can be attributed to the efficient Z-scheme charge transfer mechanism, which facilitates spatial separation of photogenerated electrons and holes while retaining their strong redox capabilities.^45^

Further, photoluminescence (PL) spectra were employed to evaluate the charge recombination behavior of the materials under irradiation. As shown in Figure 2E, the PL intensity of BiOCl/NbMo_–10_ was significantly reduced compared to that of BiOCl and NbMo_–10_, indicating effective suppression of charge carrier recombination. This result highlights the critical role of the p–n heterojunction in facilitating rapid electron separation and transfer.^46,47^ Moreover, the average lifetime of BiOCl/NbMo_–10_ was clearly extended to 6.49 ns, compared with pristine NbMo_–10_ (5.68 ns) and BiOCl (5.77 ns), as shown in Figure 2F. This enhancement can be attributed to the formation of a p–n heterojunction between BiOCl and NbMo_–10_, which benefits from their well-aligned band structures. This close alignment facilitates more efficient separation of photogenerated electron–hole pairs (e^–^–h^+^), thereby prolonging the carrier lifetime.^48^

### 3.2 Photocatalytic Degradation of BiOCl/HMo_*x*_Nb_3–*x*_O_8_ Materials

Several key parameters—including analyte pH, initial concentrations of pollutant solutions, and photocatalyst dosages—were optimized and are presented in Figures S10–S12. Based on the optimized conditions (Figure S13) and considering real wastewater scenarios, a methyl orange (MO) solution at 10 mg·L^–1^ was selected as the model pollutant to evaluate the photocatalytic performance of various BiOCl/HMo_*x*_Nb_3–*x*_O_8_ materials through photodegradation assays. Prior to the photodegradation tests, dark adsorption experiments were conducted to establish adsorption equilibrium. Figures 3A and S14 illustrate the time-dependent UV–vis absorption spectra of MO during the photodegradation process catalyzed by the different congener materials, with the corresponding photodegradation results shown in Figure 3B. The concentration of the MO solution revealed a decreasing trend with photocatalysts that offered larger surface areas, but it remained relatively high—close to its initial level—during the 30 min dark adsorption phase. This confirmed the superior adsorption capacity of materials with larger surface areas for MO molecules. Under simulated light irradiation, pure BiOCl and NbMo_–10_ displayed limited MO degradation, with removal efficiencies of 33% and 44%, respectively. However, the *in situ* chemical growth of BiOCl on HMo_*x*_Nb_3–*x*_O_8_ materials significantly enhanced the photocatalytic degradation efficiency of all the p–n heterostructured composites. Notably, the optimized BiOCl/NbMo_–10_ achieved the highest removal efficiency of 98.1% for MO, outperforming BiOCl/NbMo_–5_, BiOCl/NbMo_–10‑s_ (prepared through physical mixing), and BiOCl/NbMo_–12_ by 1.5, 1.6, and 1.1 times, respectively, within 100 min. Further, the apparent first-order reaction rate constants of these photocatalysts are presented in Figure 3C. The heterostructured BiOCl/HMo_*x*_Nb_3–*x*_O_8_ materials exhibited higher kinetic rate constants than pure BiOCl and HMo_*x*_Nb_3–*x*_O_8_. Among them, BiOCl/NbMo_–10_ showed the highest rate constant of 0.0257 min^–1^, confirming the enhanced separation efficiency of photogenerated electron–hole pairs (e^–^–h^+^), attributed to the synergistic interaction between BiOCl and HMo_*x*_Nb_3–*x*_O_8_.

**Figure 3:**
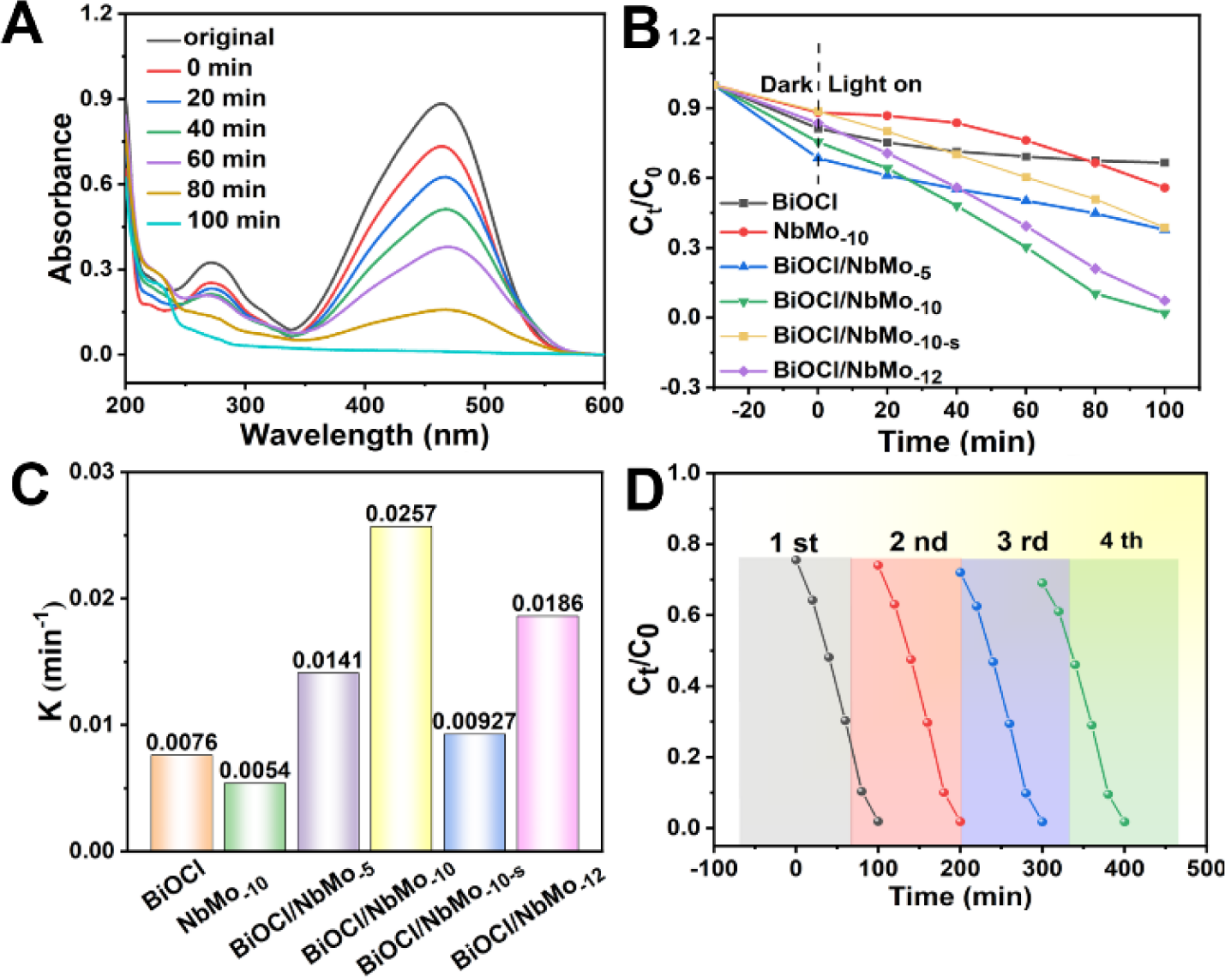
(A) Absorption spectra of a MO solution in the presence of BiOCl/NbMo_–10_ under UV–vis irradiation. (B) Effect of initial concentration of MO on its photodegradation. (C) Kinetic rate constants of different catalysts. (D) Reusability of BiOCl/NbMo_–10_ for photodegradation of MO in four consecutive cycles ([MO] = 10 mg·L^–1^).

To assess the reusability of the BiOCl/NbMo_–10_ photocatalyst, four consecutive photodegradation cycles of MO were conducted. After each cycle, the remaining catalyst was recovered by washing with water and drying for subsequent use. For each new cycle, 20 mg of the used BiOCl/NbMo_–10_ was added to a fresh MO solution (10 mg·L^–1^, 50 mL) for further degradation. As shown in Figure 3D, BiOCl/NbMo_–10_ exhibited excellent stability over four consecutive photodegradation cycles, with virtually no loss in photocatalytic activity, demonstrating good reusability for MO degradation. To further evaluate the chemical stability of BiOCl/NbMo_–10_, PXRD patterns and SEM micrographs were recorded to compare the catalyst before and after photodegradation. As presented in Figures S16C and S17, no significant changes were observed in the crystal structure or the characteristic flower-like morphology of BiOCl/NbMo_–10_. These results confirm the excellent mechanical and chemical stability of the prepared photocatalyst. Notably, the prepared BiOCl/NbMo_–10_ could easily disperse in the MO solution under magnetic stirring and precipitate rapidly within 1 min after agitation was stopped. This behavior ensures effective uptake of MO onto the catalyst surface, enhancing photocatalytic degradation, and facilitates the easy separation of sorbent from the solution, underpinning its high MO removal performance.

### 3.3 Contaminant Degradation Mechanism

To investigate the fundamental principles underlying the photocatalytic processes, a series of experiments were conducted to identify and understand the contributions of various reactive radicals. TEMPO, IPA, and EDTA-2Na were employed as general quenching agents for the active radicals ·O_2_^–^, ·OH, and h^+^, respectively. As shown in Figure 4A, the MO removal efficiency dropped to 32.68% of the initial solution upon the addition of TEMPO, indicating the significant role of ·O_2_^–^ in the degradation process. Moreover, the degradation efficiency decreased to 22.33% and 27.82% following the addition of IPA and EDTA-2Na, respectively, highlighting the crucial roles of ·OH and h^+^ in driving the photocatalytic degradation of MO molecules. The presence of free radicals during the reaction was further validated using electron spin resonance (ESR) spectroscopy. Comparisons of the ESR signals for radicals in NbMo_–10_ and BiOCl/NbMo_–10_ are shown in Figure 4B to D. The signal intensities of ·OH, h^+^, and ·O_2_^–^ radicals in BiOCl/NbMo_–10_ were observed to be significantly stronger than those in NbMo_–10_. This provides compelling evidence for the superior photocatalytic activity of BiOCl/NbMo_–10_ compared to NbMo_–10_. Furthermore, Figure S15A exhibited no discernible peaks in the absence of irradiation at 0 min. However, after exposing the BiOCl/NbMo_–10_ photocatalysts to light for 10 min, clearly identifiable signals of DMPO-·OH radicals with a characteristic ratio of 1:2:2:1 were observed, confirming the generation of ·OH under irradiation. The ESR results in Figure S15B demonstrated a decrease in signal intensity after 10 min of irradiation, likely caused by the oxidation of TEMPO via h^+^ radicals, thereby confirming the presence and involvement of h^+^ radicals in the photocatalytic degradation process. On top of this, ESR signals with a ratio of 1:1:1:1 observed after 10 min of irradiation in Figure S15C verified the involvement of ·O_2_^–^ radicals in the photocatalytic process.

**Figure 4:**
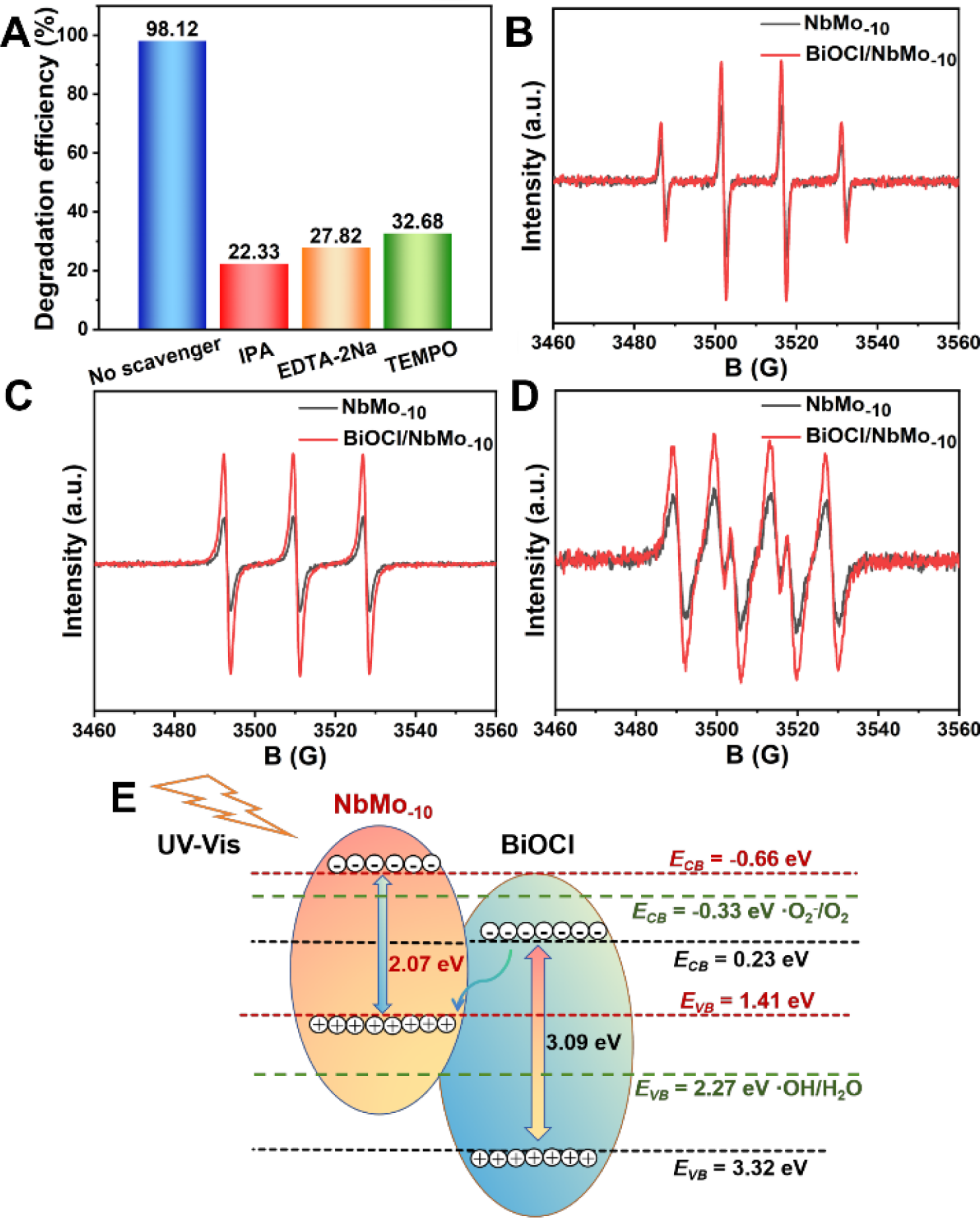
(A) Effects of different scavengers on photocatalytic degradation efficiencies. (B–D) ESR spectra of DMPO-·OH, TEMPO-h^+^ and DMPO-·O_2_^–^ over NbMo_–10_ and BiOCl/NbMo_–10_. (E) Plausible photocatalytic mechanism for BiOCl/NbMo_–10_ on degrading MO.

The enhanced photocatalytic activity of the prepared p–n heterojunction BiOCl/NbMo_–10_ catalysts likely results from cooperative interactions that promote the efficient separation of photoexcited e^–^–h^+^ pairs. Two potential electron transfer mechanisms for BiOCl/NbMo_–10_ are proposed. As illustrated in Figure 4E, one hypothesis involves the formation of a Type-II heterojunction between BiOCl and NbMo_–10_. NbMo_–10_, with a bandgap of 2.07 eV, demonstrates good visible light absorption. In contrast, BiOCl, with a bandgap of 3.09 eV, exhibits strong ultraviolet absorption but limited effectiveness in the visible range. Typically, for n-type semiconductors, the conduction band (CB) edge potential is approximately 0.1 V more negative than the Fermi level (*E*_f_), while for p-type semiconductors, the valence band (VB) edge potential is about 0.1 V more positive than the *E*_f_.^49,50^ Consequently, the E_f_ of NbMo_–10_ and BiOCl are ca. −0.54 and 3.44 eV, respectively. When NbMo_–10_ integrates with BiOCl to form a p–n junction, charge redistribution occurs to achieve equilibrium at the Fermi level. Electrons migrate from the higher Fermi level of NbMo_–10_ to the lower Fermi level of BiOCl until equilibrium is established.^51,52^ This transfer results in negatively charged BiOCl and positively charged NbMo_–10_ regions at the interface. The energy bands of NbMo_–10_ and BiOCl align, descending and ascending, respectively, to reach equilibrium with respect to the Fermi levels.^53^ An internal electric field is established at the boundary between the n-type NbMo_–10_ and p-type BiOCl, which directs the movement of photogenerated e^–^–h^+^ pairs and significantly enhances charge separation. Under irradiation, electrons in the VB of both NbMo_–10_ and BiOCl are excited, generating photoinduced e^–^–h^+^ pairs. Driven by the electric field, electrons excited in the CB of NbMo_–10_ migrate to the CB of BiOCl, while photogenerated holes move from the VB of BiOCl to that of NbMo_–10_. As a result, electrons accumulate in the CB of BiOCl, and holes are confined to the VB of NbMo_–10_. However, the CB energy (ECB) of BiOCl at 0.23 eV exceeds the potential for O_2_/·O_2_^–^ (−0.33 eV vs NHE) generation, rendering BiOCl incapable of producing ·O_2_^–^, which contradicts experimental observations.

An alternative mechanism involves the Z-scheme heterojunction. In this scenario, the composite material generates e^–^–h^+^ pairs upon photon absorption. It is proposed that electrons in the CB of BiOCl recombine with holes in the VB of NbMo_–10_ at the heterojunction boundary due to their proximity. The VB energy (EVB) of BiOCl (3.32 eV) exceeds the ·OH/H_2_O potential (2.27 eV vs NHE), enabling holes in the VB of BiOCl to oxidize OH^–^ and generate ·OH radicals. Conversely, the CB energy (ECB) of NbMo_–10_ (−0.66 eV) is below the O_2_/·O_2_^–^ potential, allowing photogenerated electrons in NbMo_–10_ to efficiently reduce O_2_ to ·O_2_^–^ radicals. The Z-scheme heterojunction between BiOCl and NbMo_–10_ aligns with the fundamental principles governing free radical production, significantly reducing the recombination rate of photoexcited e^–^–h^+^ pairs. The generated free radicals subsequently degrade MO molecules, ensuring effective photocatalytic performance.

## 4 Conclusions

In this study, 2D/2D p–n heterojunction BiOCl/HMo_*x*_Nb_3–*x*_O_8_ materials were constructed using a novel *in situ* chemical absorption and hydrolysis strategy. BiOCl nanosheets were tightly integrated with the flower-like structure of HMo_*x*_Nb_3–*x*_O_8_, resulting in a large surface area, coupled with enhanced charge and mass transfer. The resulting BiOCl/HMo_*x*_Nb_3–*x*_O_8_ materials demonstrated superior photocatalytic performance for the degradation of MO under UV–vis light compared to pure BiOCl and HMo_*x*_Nb_3–*x*_O_8_ materials. The removal efficiency of the optimized catalyst (BiOCl/NbMo_–10_) was 2.94 and 2.22 times higher than that of pristine BiOCl and NbMo_–10_, respectively. Moreover, the BiOCl/NbMo_–10_ materials exhibited excellent reusability, maintaining high photocatalytic activity after four consecutive photodegradation cycles. The enhanced photocatalytic performance was confirmed through photoluminescence and photoelectrochemical analyses, attributed to the synergistic effect of the tightly integrated p–n heterojunctions formed by the *in situ* synthesis approach and the improved separating and transferring efficiency of photoinduced e^–^–h^+^. To this end, the plausible degradation mechanism was explored through active radical measurements, confirming the contributions from ·OH, h^+^, and ·O_2_^–^ radicals to MO degradation. This study offers a clear insight into the rational design and *in situ* construction of high-performance heterojunction photocatalysts, with high potential for translation into environmental remediation applications.

## Supplementary Material



## Notes

Characterizations of materials synthesis, photodegradation optimization, stability experiments of photocatalysts, and mechanism investigations (PDF)
