# Supplementary material for: In Situ Engineering of Two-Dimensional Heterostructures for Enhanced Photocatalytic Decontamination of Methyl Orange
Source: ACS Appl Eng Mater. 2025 May 14;3(5):1292–301. doi: 10.1021/acsaenm.5c00134 (PMC12104971; doi:10.1021/acsaenm.5c00134)
Supplement: Supplementary file 1 [file em5c00134_si_001.pdf]

Supporting information

In Situ Engineering of Two-dimensional Heterostructures for Enhanced Photocatalytic Decontamination of Methyl Orange

Junli Chen,[a] Xinyi Jin,[a] Pengcheng Zhang,[a] Nan Song,[a] Pan Gao,[b,*] Roland A Fischer,[b] Soumya Mukherjee[c,*]

[a] College of Materials and Chemical Engineering, Collaborative Innovation Center of Environmental Pollution Control and Ecological Restoration, Zhengzhou University of Light Industry, Zhengzhou 450002, P. R. China.

[b] Chair of Inorganic and Metal-Organic Chemistry, Department of Chemistry, School of Natural Sciences and Catalysis Research Center, Technische Universität München, Lichtenbergstraße 4, 85748 Garching b. München, Germany.

[c] Department of Chemical Sciences, Bernal Institute and Research Ireland Centre for Pharmaceuticals (SSPC), University of Limerick, Limerick V94 T9PX, Ireland.

*Corresponding author contact: chem.pan.gao@tum.de; soumya.mukherjee@ul.ie

Content

Supporting Information Figures....S3

Fig. S1 SEM micrographs of BiOCl/NbMo-10 materials S3

Fig. S2 TEM image of BiOCl/NbMo-10. ....S3

Fig. S3 XPS spectra of different catalysts.....S3

Fig. S4 BET spectra of different catalysts....S4

Fig. S5 UV-Vis diffuse reflectance spectra S4

Fig. S6 UV-Vis diffuse reflectance spectra S5

Fig. S7 VB XPS. S5

Fig. S8 Transient current responses to on-off cycles of illumination and EIS Nyquist plots. S5

Fig. S9 Photoluminescence spectra....S6

Fig. S10 UV-Vis spectra of BiOCl/NbMo-10 photocatalytic degradation of MO solutions with different pH monitored under UV-Vis irradiation....S6

Fig. S11 UV-Vis spectra of BiOCl/NbMo-10 photocatalytic degradation of different concentrations of MO solution monitored under UV-Vis irradiation....S6

Fig S12 UV-Vis spectra of BiOCl/NbMo-10 photocatalytic degradation of different concentrations of MO solution monitored under UV-Vis irradiation....S7

Fig. S13 Effect of MO solutions with different conditions on photodegradation....S7

Fig. S14 UV-Vis spectra of MO solution monitored under irradiation…………… S8

Fig. S15 ESR spectra of BiOCl/NbMo-10....S8

Fig. S16 XRD comparison before and after reaction with different catalysts. S8

Fig. S17 SEM micrographs after reaction....S9

Supporting Information Tables S9

Table S1 BET surface areas, pore volume, and average pore size of different photocatalysts. S9

Table S2 The fitted EIS resistance S9

Table S3 Fluorescence lifetime composition S9

Table S4 Comparison of MO degradability using different photocatalysts.....S10

References....S10

Supporting Information Figures

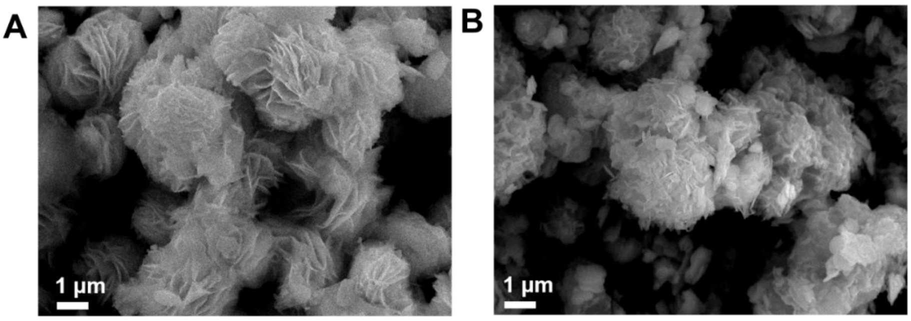

Fig. S1 SEM micrographs of BiOCl/NbMo-10 materials (A) before washing with H_2O and (B) after washing with H_2O.

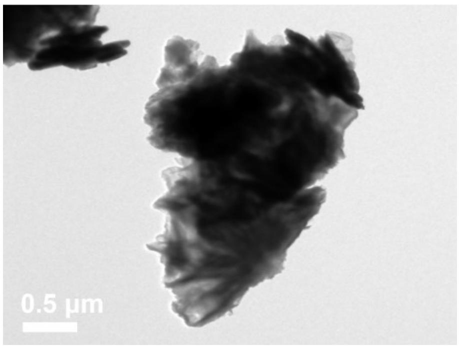

Fig. S2 TEM image of BiOCl/NbMo-10.

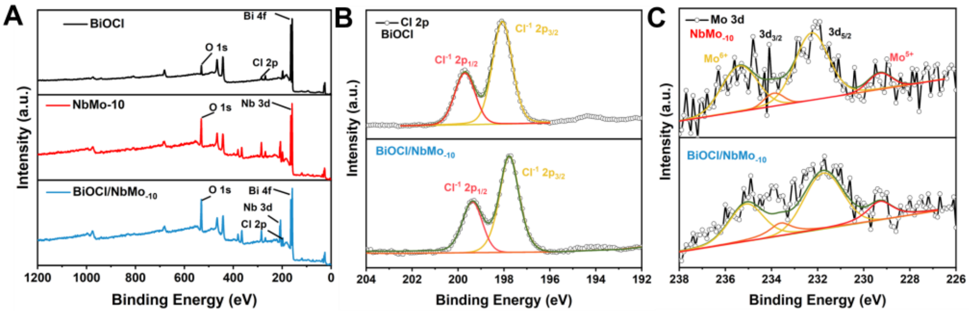

Fig. S3 XPS spectral signatures for (A) BiOCl, NbMo-10, BiOCl/NbMo-10; (B) Cl 2p; (C) Mo 3d.

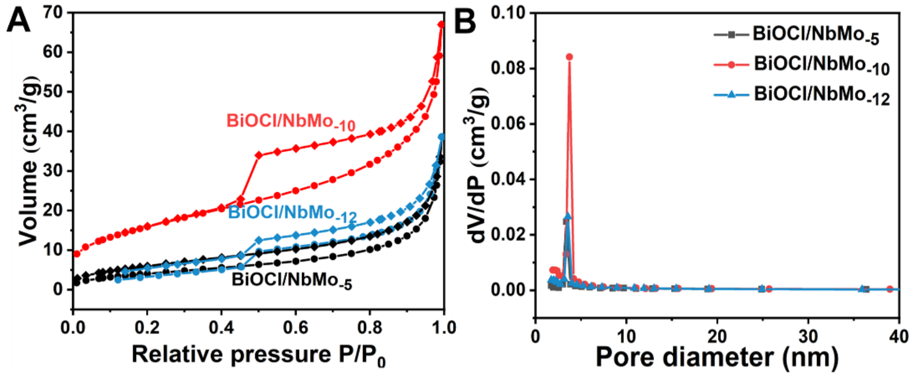

Fig. S4 (A) N_2 adsorption-desorption isotherms of BiOCl/NbMo-5, BiOCl/NbMo-10 and BiOCl/NbMo-12, (B) pore size distribution of BiOCl/NbMo-5, BiOCl/NbMo-10 and BiOCl/NbMo-12.

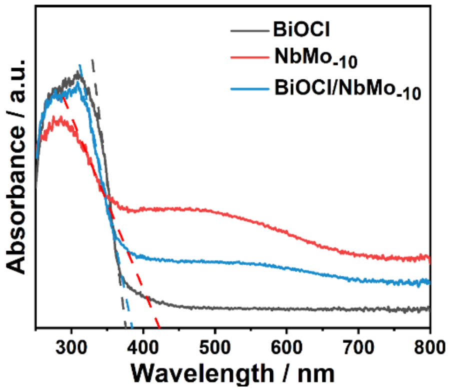

Fig. S5 UV-Vis diffuse reflectance spectra of BiOCl, NbMo-10 and BiOCl/NbMo-10 materials.

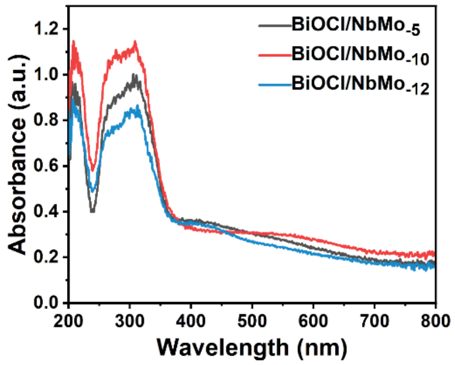

Fig. S6 UV-Vis diffuse reflectance spectra of BiOCl/NbMo$_{-5}$, BiOCl/NbMo$_{-10}$ and BiOCl/NbMo$_{-12}$ materials.

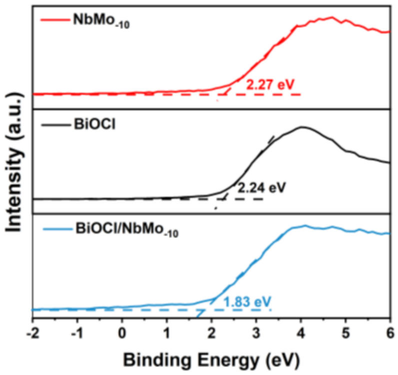

Fig. S7 VB XPS of NbMo$_{-10}$, BiOCl, BiOCl/NbMo$_{-10}$.

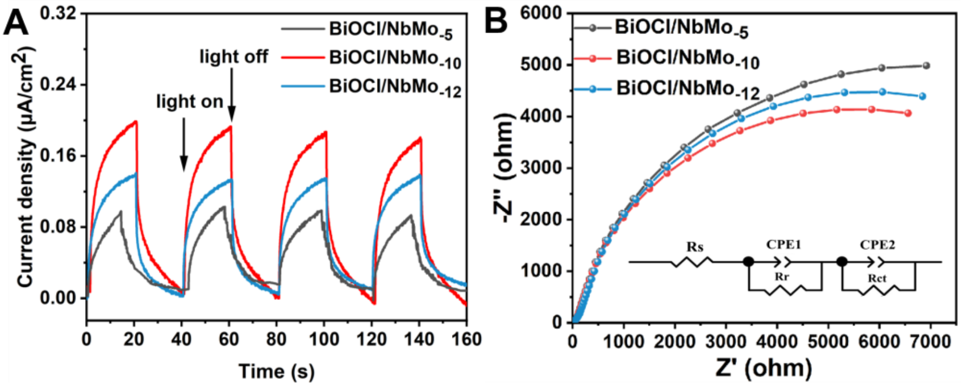

Fig. S8 (A) Transient current responses to on-off cycles of illumination on BiOCl/NbMo$_{-5}$, BiOCl/NbMo$_{-10}$ and BiOCl/NbMo$_{-12}$, (B) EIS Nyquist plots of BiOCl/NbMo$_{-5}$, BiOCl/NbMo$_{-10}$ and BiOCl/NbMo$_{-12}$ at open circuit voltage.

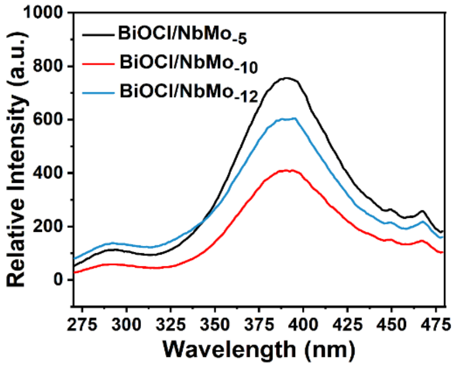

Fig. S9 Photoluminescence spectra of BiOCl/NbMo-5, BiOCl/NbMo-10, and BiOCl/NbMo-12 materials.

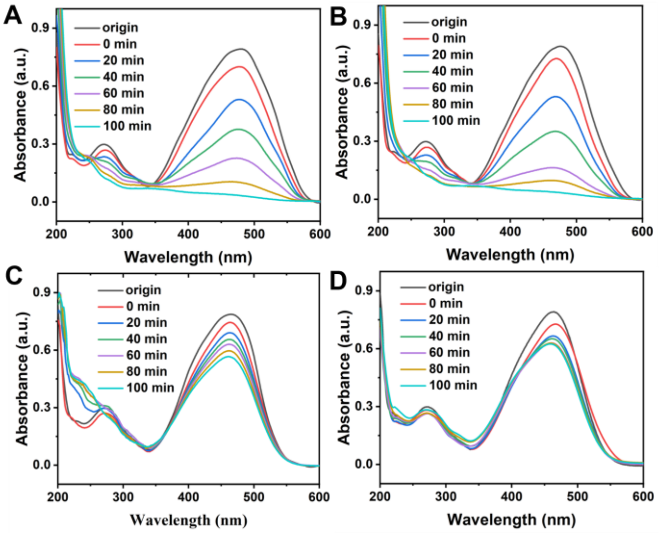

Fig. S10 UV-Vis spectra of BiOCl/NbMo-10 photocatalytic degradation of MO solutions with different pH monitored under UV-Vis irradiation: (A) PH=4; (B) PH-5; (C) PH=7; (D) PH=8.

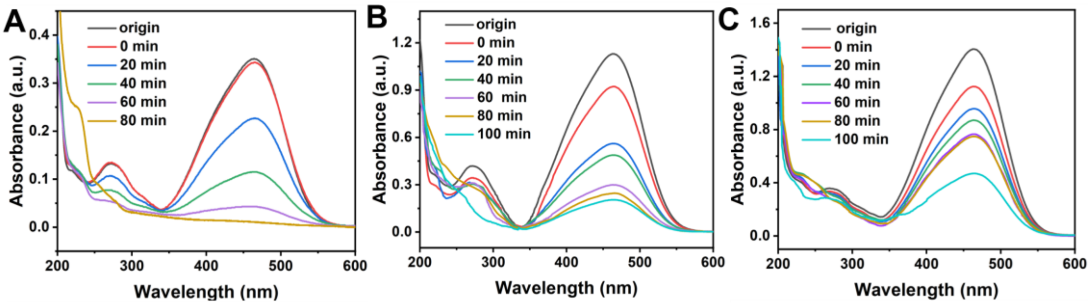

Fig. 11 UV-Vis spectra of BiOCl/NbMo-10 photocatalytic degradation of different concentrations of MO solution monitored under UV-Vis irradiation: (A) 5 mg·L^{-1}; (B) 15 mg·L^{-1}; (C) 20 mg·L^{-1}.

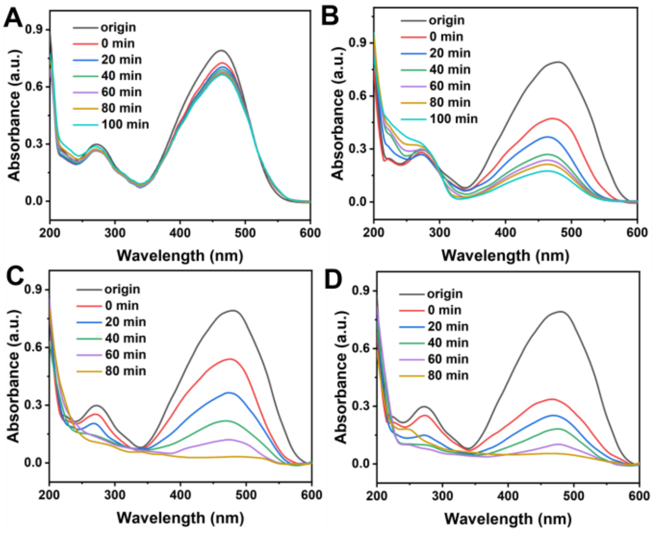

Fig S12 UV-Vis spectra of BiOCl/NbMo-10 photocatalytic degradation of different concentrations of MO solution monitored under UV-Vis irradiation: (A) 5 mg; (B) 10 mg; (C) 30 mg; (D) 50 mg.

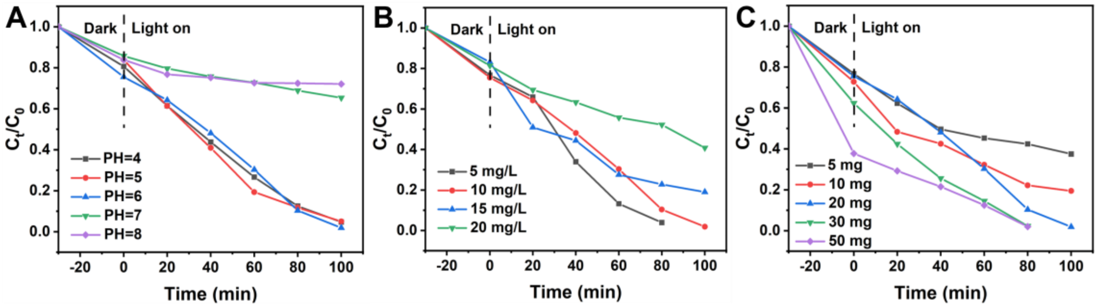

Fig. S13 (A) Effect of MO solutions with different initial pH on photodegradation; (B) Effects of MO solutions with different initial concentrations on photodegradation; (C) Effect of different quality of BiOCl/NbMo-10 on photodegradation.

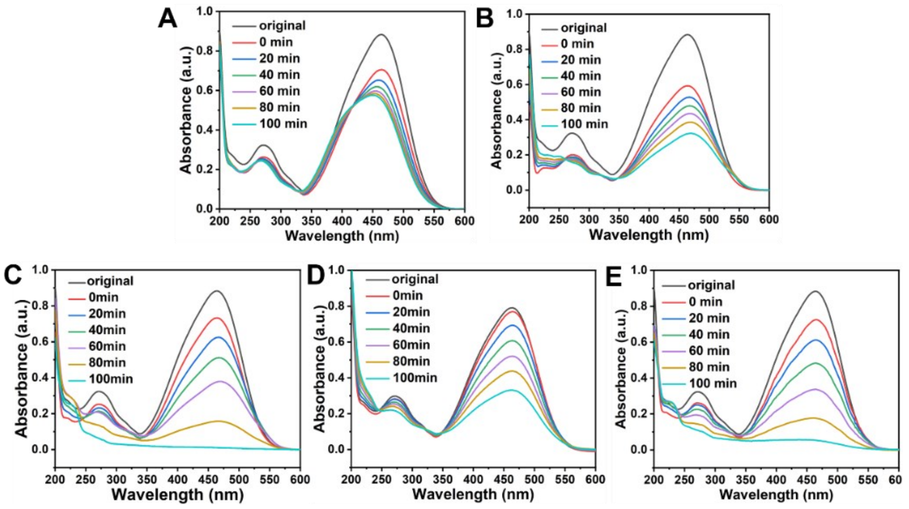

Fig. S14 UV-Vis spectra of MO solution monitored under UV-Vis light irradiation treated by (A) BiOCl, (B) BiOCl/NbMo$_{-5}$, (C) BiOCl/NbMo$_{-10}$, (D)BiOCl/NbMo-10-s, (E) BiOCl/NbMo$_{-12}$.

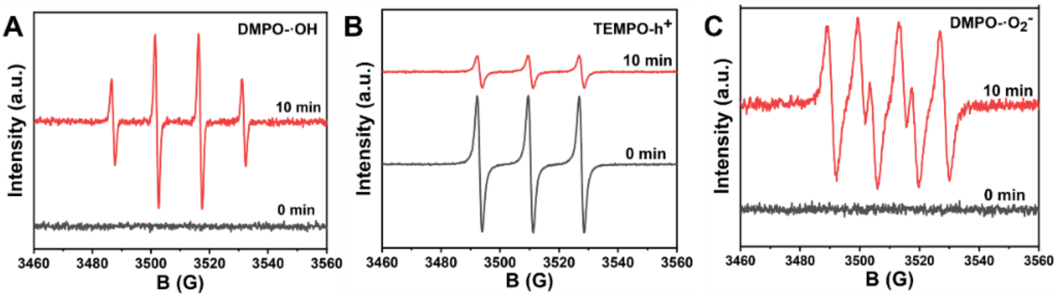

Fig. S15 ESR spectra of BiOCl/NbMo$_{-10}$ for the signal of (A) DMPO- ·OH, (B) TEMPO-h$^+$, (C) DMPO- ·O_2^-.

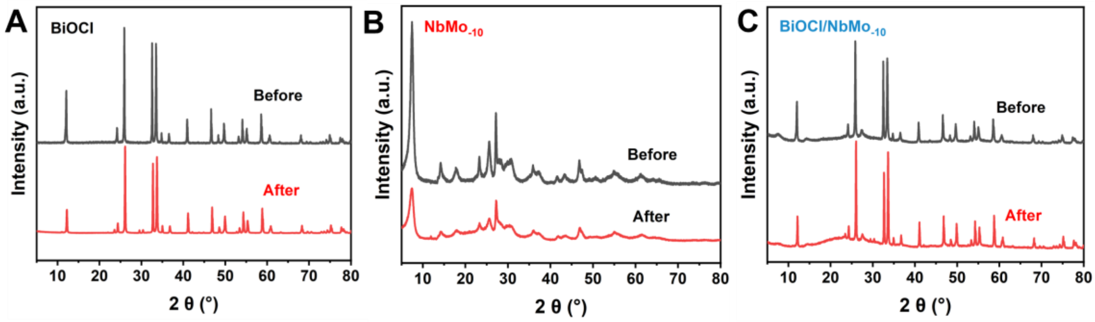

Fig. S16 (A)-(C) XRD comparison before and after reactions with the different catalysts.

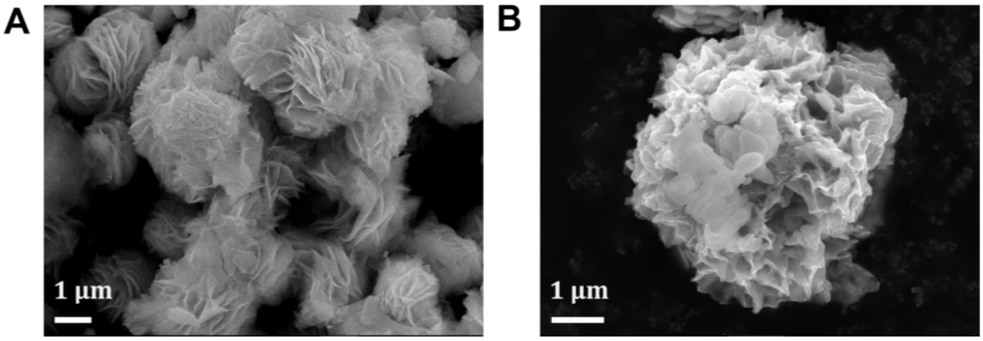

Fig. S17 SEM micrographs recorded post-reaction: (A) $NbMo_{-10}$; (b) BiOCl/$NbMo_{-10}$.

Supporting Information Tables

Table S1 BET surface areas, pore volume, and average pore size of different photocatalysts.

sample	BET surface area ($m^2 \cdot g^{-1}$)	Pore volume ($cm^3 \cdot g^{-1}$)	Average pore size (nm)
BiOCl	4.97	0.026	25.41
$NbMo_{-10}$	9.35	0.045	15.44
BiOCl/$NbMo_{-5}$	17.88	0.061	9.73
BiOCl/$NbMo_{-10}$	58.07	0.11	6.04
BiOCl/$NbMo_{-12}$	25.26	0.062	7.70

Table S2 The fitted EIS resistances of BiOCl, $NbMo_{-10}$, BiOCl/$NbMo_{-5}$, BiOCl/$NbMo_{-10}$, BiOCl/$NbMo_{-12}$.

Sample	R_s (Ω)	R_r (Ω)	R_{ct} (Ω)
BiOCl	14.90	1534	133.20
$NbMo_{-10}$	9.96	1183	117.20
BiOCl/$NbMo_{-5}$	12.59	1525	127.20
BiOCl/$NbMo_{-10}$	13.90	1341	109
BiOCl/$NbMo_{-12}$	14.61	1816	158

Table S3 Fluorescence lifetime compositions of BiOCl, $NbMo_{-10}$, BiOCl/$NbMo_{-10}$.

**Sample**	**τ_1 (ns)**	**A_1**	**τ_2 (ns)**	**A_2**	**τ_a (ns)**
BiOCl	2.08	508.56	10	88.11	5.68
$NbMo_{-10}$	2.09	458.3	10	83.28	5.77
BiOCl/$NbMo_{-10}$	2.10	457.14	10.43	102.87	6.49

Table S4 Comparison of MO degradability using different photocatalysts, *vs.* BiOCl/$HMo_xNb_{3-x}O_8$.

Photocatalyst	Catalyst concentration (mg mL^{-1})	MO concentration (mg L^{-1})	Removal efficiency	Removal time (min)	Ref.
$CuCo_2O_4$	1.0	5	85 %	180	[1]
Ag-AgI/Bi	0.4	20	61 %	180	[2]
g-C_3N_4@ZnO	1.0	3	91.2 %	120	[3]
$CeVO_4$/BiVO4/rGO	0.4	10	90 %	120	[4]
ZnO (Ag@ZnO)	0.5	19.4	100 %	120	[5]
M-008 MOF	0.5	100	95 %	90	[6]
Ag/MO_3/TiO_2	1.0	10	96.5 %	300	[7]
BiOCl/$HMo_xNb_{3-x}O_8$	0.4	10	98.1 %	100	This work

References

[1]. L. Gnanasekaran; D. Shanmugapriya; V. Sasikala; S. Vadivel; W.-H. Chen; M. Arthi; M. Soto-Moscoso, Nanocubic copper cobaltite for methyl orange degradation through photocatalytic process. Chemosphere, 312 (2023), 137311.

[2]. B. Xu; Y. Li; Y. Gao; S. Liu; D. Lv; S. Zhao; H. Gao; G. Yang; N. Li; L. Ge, Ag-AgI/Bi_3O_4Cl for efficient visible light photocatalytic degradation of methyl orange: The surface plasmon resonance effect of Ag and mechanism insight, Appl. Catal., B: Environmental, 246 (2019), 140-148.

[3]. C. Lin; J. Su; Z. Chen; S. Zhang; Q. Gong; Y. Qu; J. Fei; X. Ye; J. Zhang, Photocatalytic oxidative degradation of methyl orange by a novel g-C_3N_4@ZnO based on graphene oxide composites with ternary heterojunction construction, React. Kinet., Mech. Catal, 135 (2022), 1651-1664.

[4]. S. Pu; L. Xu; L. Sun; H. Du, Tuning the optical properties of the zirconium–UiO-66 metal–organic framework for photocatalytic degradation of methyl orange. Inorg. Chem. Commun, 52 (2015), 50-52.

[5]. V. A. Tran; A. N. Kadam; S.-W. Lee, Adsorption-assisted photocatalytic degradation of methyl orange dye by zeolite-imidazole-framework-derived nanoparticles. J. Alloys Compd, 835 (2020), 155414.

[6]. L. A. Alfonso-Herrera; A. M. Huerta-Flores; L. M. Torres Martínez; D. J. Ramírez-Herrera; J. M. Rivera-Villanueva, M-008: A stable and reusable metalorganic framework with high crystallinity applied in the photocatalytic hydrogen evolution and the degradation of methyl orange. J. Photochem. Photobiol., A, 389 (2020), 112240.

[7]. S. Kader; M. R. Al-Mamun; M. B. K. Suhan; S. B. Shuchi; M. S. Islam, Enhanced photodegradation of methyl orange dye under UV irradiation using MoO_3 and Ag doped TiO_2 photocatalysts. Environ. Technol. Innovation, 27 (2022), 102476.
